# The Stiff Elbow Revisited: How Much Motion Can Surgery Really Restore?

**DOI:** 10.3390/medicina62091658

**Published:** 2026-08-29

**Authors:** Mihai Tudor Gavrila, Traian Eduard Stanescu, Dinu Gartonea

**Affiliations:** Orthopadie, Emergency Hospital Saint Pantelimon Bucharest, 021659 Bucuresti, Romania; stanescu.teduard94@gmail.com (T.E.S.); dinumed@gmail.com (D.G.)

**Keywords:** stiff elbow, flexion–extension arc, arthrolysis, triceps tenolysis, ulnar transposition

## Abstract

*Background and Objectives*: Post-traumatic elbow stiffness is a disabling condition often resulting from a combination of capsular contracture, osseous impingement, and residual instability, rendering it exceptionally challenging to treat. The aim of this study was to evaluate the outcomes of a comprehensive surgical approach for the treatment of post-traumatic elbow stiffness. *Materials and Methods*: A retrospective case series of 20 consecutive patients treated between 2018 and 2025 was conducted. Etiologies included distal humeral fractures (*n* = 9), terrible triad injuries (*n* = 4), elbow dislocations (*n* = 3), olecranon fractures (*n* = 2), and neglected radial head fractures (*n* = 2). All patients underwent anterior and posterior arthrolysis with triceps tenolysis. Additional procedures included ligament reconstruction, radial head arthroplasty, anterior capsule repair, tricepsplasty, and ulnar nerve transposition when indicated. Elbow range of motion was assessed preoperatively and during follow-up at 2 weeks, 6 weeks, 3 months, 6 months, and 1 year postoperatively. *Results*: Mean flexion improved from 81.0° to 125.3°, while the extension deficit improved from 66.5° to 16.5°. Mean supination increased from 54.8° to 82.8°, and mean pronation from 67.0° to 84.5°. The mean flexion–extension arc improved from 14.5° to 108.8°, representing a mean gain of 94.3°. One transient sensory ulnar neuropathy and one case of postoperative triceps insufficiency requiring revision surgery were observed; one patient developed postoperative skin blistering following a posterior approach. *Conclusions*: Comprehensive, pathology-oriented surgical treatment resulted in substantial improvement in elbow motion and restoration of a functional range of movement.

## 1. Introduction

Traumatic elbow injuries represent some of the most challenging pathologies to treat due to the combination of capsular contracture, ligament retraction, osseous impingement, and residual instability. Loss of motion following trauma may severely compromise upper-limb function, affecting activities of daily living, work-related tasks, and overall quality of life. Despite advances in fracture fixation techniques and rehabilitation protocols, elbow stiffness remains a frequent complication after fractures and dislocations around the elbow (an incidence of 10–15%) [1].

A functional elbow has traditionally been defined as a flexion–extension arc between 30° and 130° and a forearm rotation arc of at least 100° (50° in both supination and pronation) [2]. However, many patients fail to regain this functional range following trauma, particularly after complex injuries requiring surgical treatment. Distal humeral fractures, terrible triad injuries, elbow dislocations, olecranon fractures, and radial head fractures are among the most common causes of post-traumatic stiffness.

The pathogenesis of elbow stiffness is characteristically multifactorial rather than isolated to a single etiology. Based on the anatomical site of the contracture, Morrey categorized the condition into three distinct groups: intrinsic (driven by intra-articular adhesions, cartilage degradation, retained hardware, or articular malunions), extrinsic (encompassing capsular-ligamentous fibrotic changes, extra-articular malunions, and heterotopic ossification), and combined forms [3]. Pathophysiologically, the evolution of post-traumatic joint restriction undergoes a predictable four-stage cascade outlined by O’Driscoll: hemorrhage, edema, granulation tissue formation, and dense tissue fibrosis. Clinically, this rigid state frequently manifests as the cumulative consequence of suboptimal initial stabilization, deferred physical therapy, prolonged postoperative immobilization, or unaddressed structural injuries [4].

Several multicenter studies have demonstrated that stable osteosynthesis allowing early postoperative mobilization significantly decreases the incidence of post-traumatic elbow stiffness [5,6,7,8]. In addition, splinting, physiotherapy, and both corticosteroids [9] and nonsteroidal anti-inflammatory drugs [10] have been reported to reduce the likelihood of developing this complication. Numerous surgical techniques have been described for the treatment of the stiff elbow, including open or arthroscopic approaches [11,12,13]. However, restoration of motion often necessitates simultaneous management of capsular contracture, triceps adhesions, ligament insufficiency, radial head pathology, ulnar nerve involvement, and residual osseous abnormalities [14].

The purpose of this study was to evaluate the clinical and functional outcomes of a comprehensive, pathology-oriented reconstructive surgical strategy in patients presenting with complex post-traumatic elbow stiffness. By tailoring specific open exposures and adjunctive structural procedures to each patient’s distinctive anatomy, we aimed to demonstrate that a customized reconstructive approach can significantly improve multi-planar mobility, maximize pain relief, and successfully restore a functional range of motion.

## 2. Materials and Methods

### 2.1. Study Design

This study was designed as a retrospective consecutive case series evaluating the clinical and functional outcomes of surgical management in patients with post-traumatic elbow stiffness. The study included all eligible patients treated between January 2018 and December 2025 at a single tertiary orthopedic center. All surgical procedures were performed by the senior author, ensuring consistency in surgical indication, operative technique, and postoperative management.

The study was conducted in accordance with the principles of the Declaration of Helsinki and institutional ethical standards. Ethical approval was obtained from the local institutional review board, and all patients provided informed consent for surgical treatment and use of anonymized clinical data for research purposes.

Patients were included if they presented with clinically significant post-traumatic elbow stiffness associated with functional impairment in activities of daily living (an extension deficit greater than 30°, flexion less than 120°, or a forearm rotational arc of less than 100°) [2] and failure of a structured conservative treatment protocol, including supervised physiotherapy [15]. Functional limitation was defined as restriction in daily activities attributable to loss of elbow flexion–extension and/or forearm rotation.

Exclusion criteria included active or previous deep infection of the elbow, incomplete clinical or radiological follow-up, and cases in which stiffness was not primarily post-traumatic in origin (e.g., inflammatory arthropathy, neurological contractures, or congenital conditions).

### 2.2. Preoperative Assessment

All patients underwent a standardized preoperative evaluation including detailed clinical and imaging assessment. Clinical examination included measurement of active and passive ROM in flexion–extension and pronation-supination, recorded using a calibrated goniometer. Pain, joint stability, and functional limitations were systematically documented. Neurological evaluation focused on ulnar nerve symptoms, including sensory changes and motor deficits.

**Figure 1:**
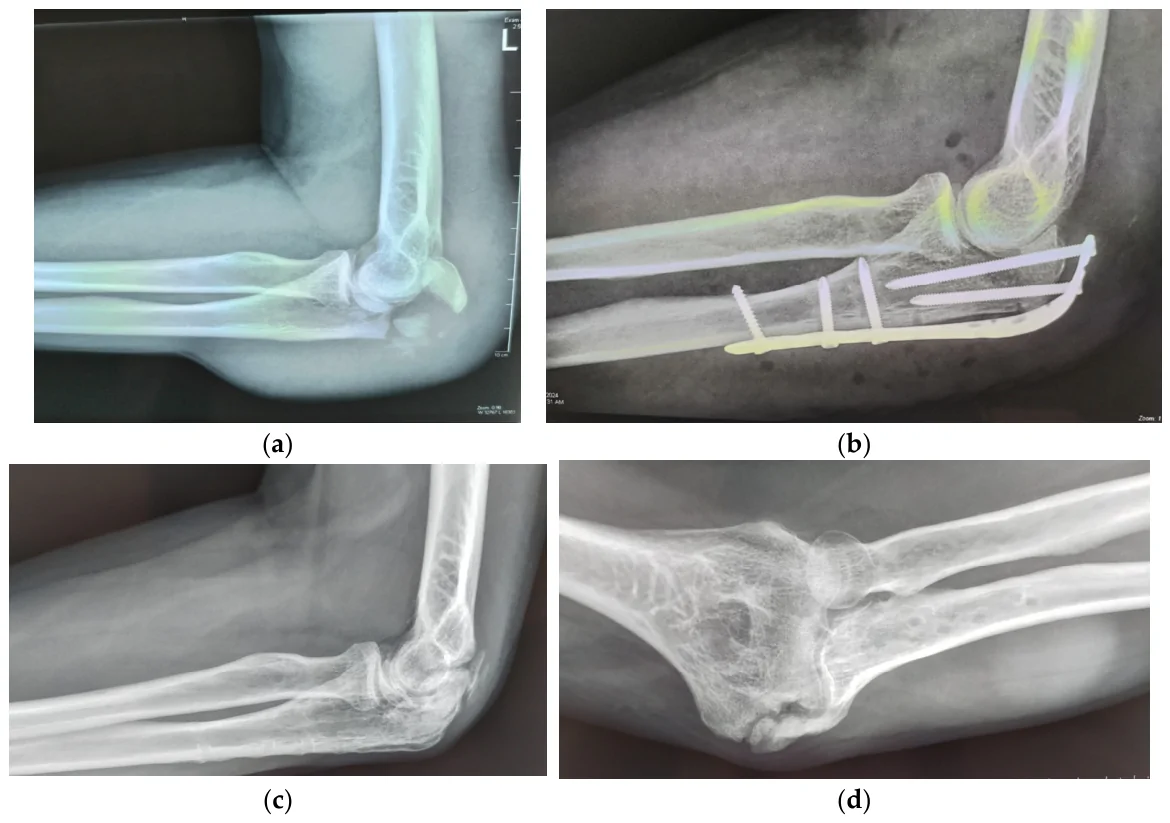

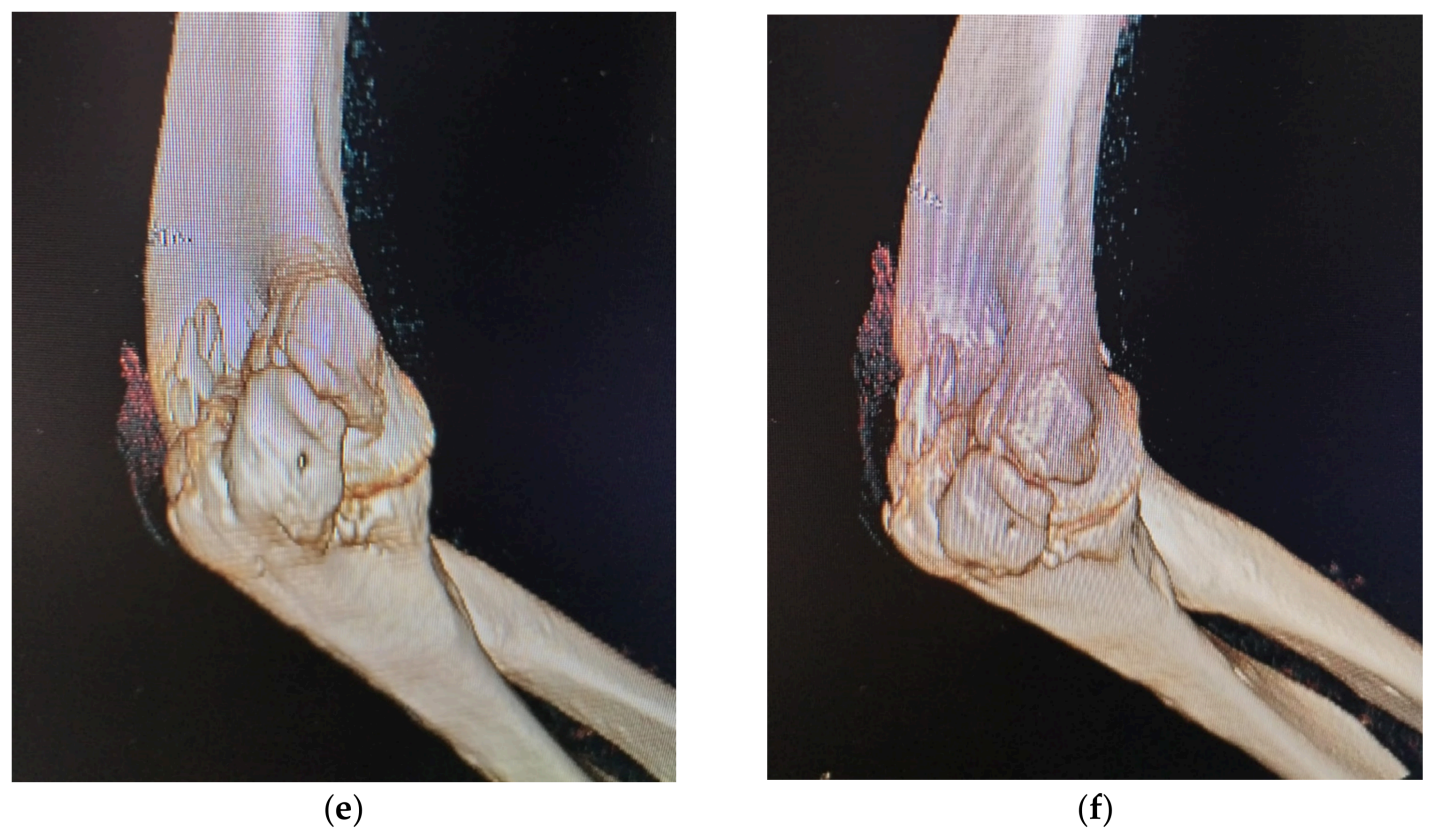
X-ray (**a**) fracture of olecranon, (**b**) osteosynthesis with a plate and screws, (**c**,**d**) secondary periarticular ossification, (**e**,**f**) CT findings.

Radiographic evaluation included standard anteroposterior and lateral views of the elbow in all patients. Computed tomography (CT) was systematically performed to assess osseous anatomy, including malunion, intra-articular incongruity, osteophytes, heterotopic ossification, retained hardware, and mechanical impingement (Figure 1). Magnetic resonance imaging (MRI) was selectively used in cases where soft tissue pathology was suspected, particularly ligament insufficiency, cartilage injury, or complex capsuloligamentous fibrosis.

### 2.3. Outcome Measures

Clinical outcomes were assessed using both objective and functional parameters. The primary outcome measure was improvement in elbow range of motion (ROM), specifically flexion–extension arc and forearm pronation-supination. Secondary outcome measures included Functional scores using the **Mayo Elbow Performance Score (MEPS),** Disability assessment using the **Disabilities of the Arm, Shoulder and Hand (DASH)** questionnaire, Pain evaluation using a Visual Analog Scale (VAS), assessment of ulnar nerve function and elbow stability. Range of motion was measured using a standardized universal goniometer by independent examiners not directly involved in the surgical procedures, when available.

### 2.4. Surgical Technique and Algorithmic Decision-Making

In the present series, in accordance with recommendations in the relevant literature and following the formal documentation of a failed structured conservative physical therapy protocol, all patients underwent surgery between 6 and 12 months after the initial traumatic event [16,17].

The surgical approach was individualized based on preoperative clinical findings, the localization of dominant structural blocks, and prior surgical scars. The selection criteria for the specific surgical exposures were standardized as follows:

- **Lateral Approach:** Indicated for extrinsic or combined contractures presenting with predominantly anterior and lateral pathology (e.g., radiocapitellar arthritis or radial head malunions), a clinically preserved triceps mechanism, a complete absence of ulnar nerve symptoms, or valgus instability. The lateral approach is also indicated if the patient presents with preoperative varus instability, which requires lateral collateral ligament reconstruction.
- **Combined Medial-Lateral Approach:** Utilized when a comprehensive global release could not be successfully achieved via an isolated lateral incision, particularly in severe flexion contractures requiring extensive anterior capsule resection, or when medial-sided osseous impingement, coronoid osteophytes, ulnar compression, or localized heterotopic ossification required direct visualization, while deliberately preserving the structural integrity of the triceps tendon. Additionally, the medial approach is extremely useful when reconstruction of the collateral ligaments is required. The decision to utilize these approaches is dictated by the instability present preoperatively (which becomes even more apparent under anesthesia) and the associated pathology (heterotopic ossification, subluxations, etc.).
- **Posterior Approach:** Reserved for highly complex, multi-column global stiffness (e.g., severe distal humeral fracture malunions), cases with clinically significant preoperative triceps shortening or posterior soft-tissue tethering requiring a V-shaped tricepsplasty, or when circumferential exposure of both columns and wide joint debridement were necessary (Table 1).

It is essential to emphasize that the proposed surgical decision-making framework is designed not as a rigid protocol, but as a nuanced, adaptable guideline. Post-traumatic elbow contractures present distinct structural complexities that require the orthopedic surgeon to possess complete technical mastery over all available exposures—whether lateral, medial, or posterior. The final intraoperative strategy must remain highly flexible, allowing the surgeon to seamlessly adapt to patient-specific anatomical variations, the true extent of tissue fibrosis unveiled after debridement, or unpredicted intraoperative findings. Ultimately, the algorithm serves as an orientative baseline, while the surgeon’s clinical judgment and adaptive expertise dictate the tailored execution required to secure a stable and mobile joint.

The lateral approach involved dissection between the extensor carpi radialis longus and extensor carpi radialis brevis tendons, providing direct access to the anterior and lateral aspects of the elbow. Furthermore, it offered excellent exposure of the radiocapitellar joint, allowing both anterior and posterior arthrolysis to be performed through a single incision [18]. The forearm was maintained in pronation throughout the deep dissection to protect the posterior interosseous nerve. In cases requiring lateral collateral ligament reconstruction, the Kocher interval between the anconeus and extensor carpi ulnaris was used to facilitate adequate exposure and graft passage. For reconstruction of the lateral collateral ligament, PushLock^®^ anchors (Arthrex, Naples, FL, USA) were used. In one case, the neoligament was created using a previously harvested gracilis tendon, while in the other case, FiberTape^®^ (Arthrex) was utilized.

**Figure 2:**
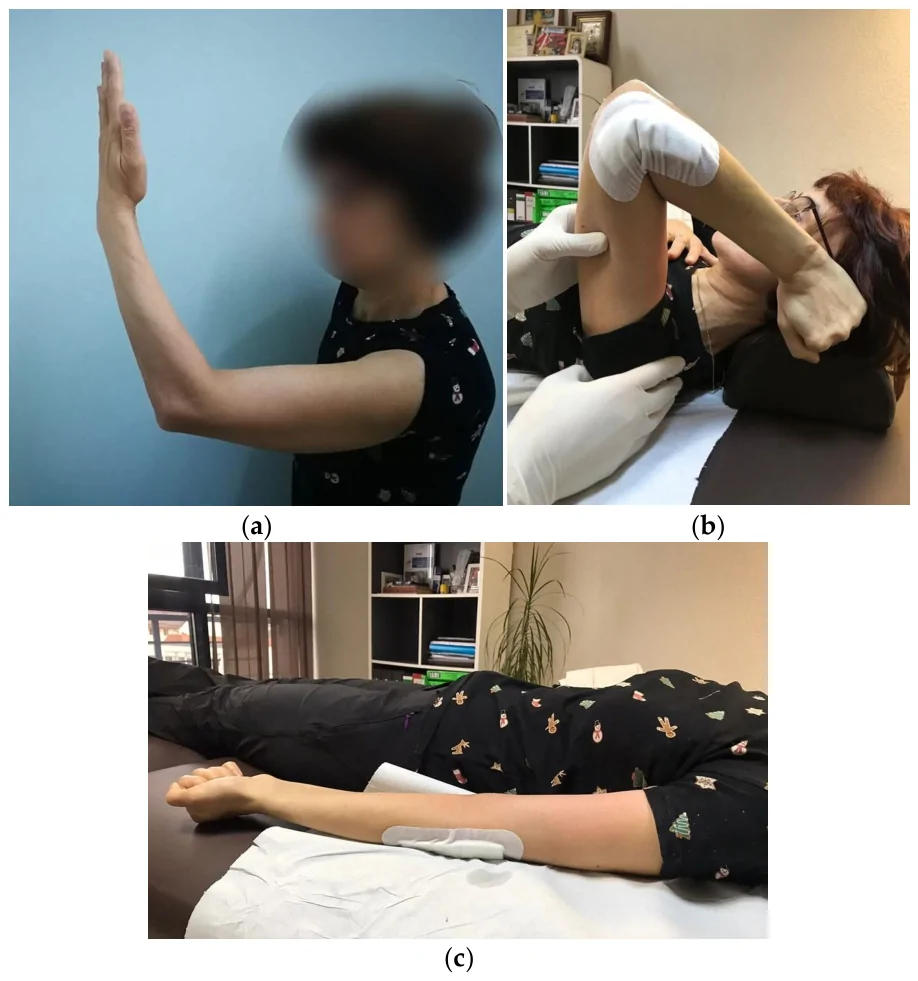
(**a**) Elbow stiffness at 90 degrees of flexion–extension following a conservatively treated radial head fracture. (**b**,**c**) Postoperative result after lateral approach.

In most cases, a lateral approach provides adequate exposure for performing arthrolysis with very good results (Figure 2). This technique has been associated with high patient satisfaction rates and improved elbow range of motion, largely due to preservation of the lateral collateral ligament (when ligament reconstruction was not required). Subsequently, the origins of the extensor carpi radialis longus and brachioradialis muscles are detached from the lateral distal column of the humerus, allowing access to and release of the anterolateral capsule. At the end of the procedure, the extensor musculature was reattached.

**Figure 3:**
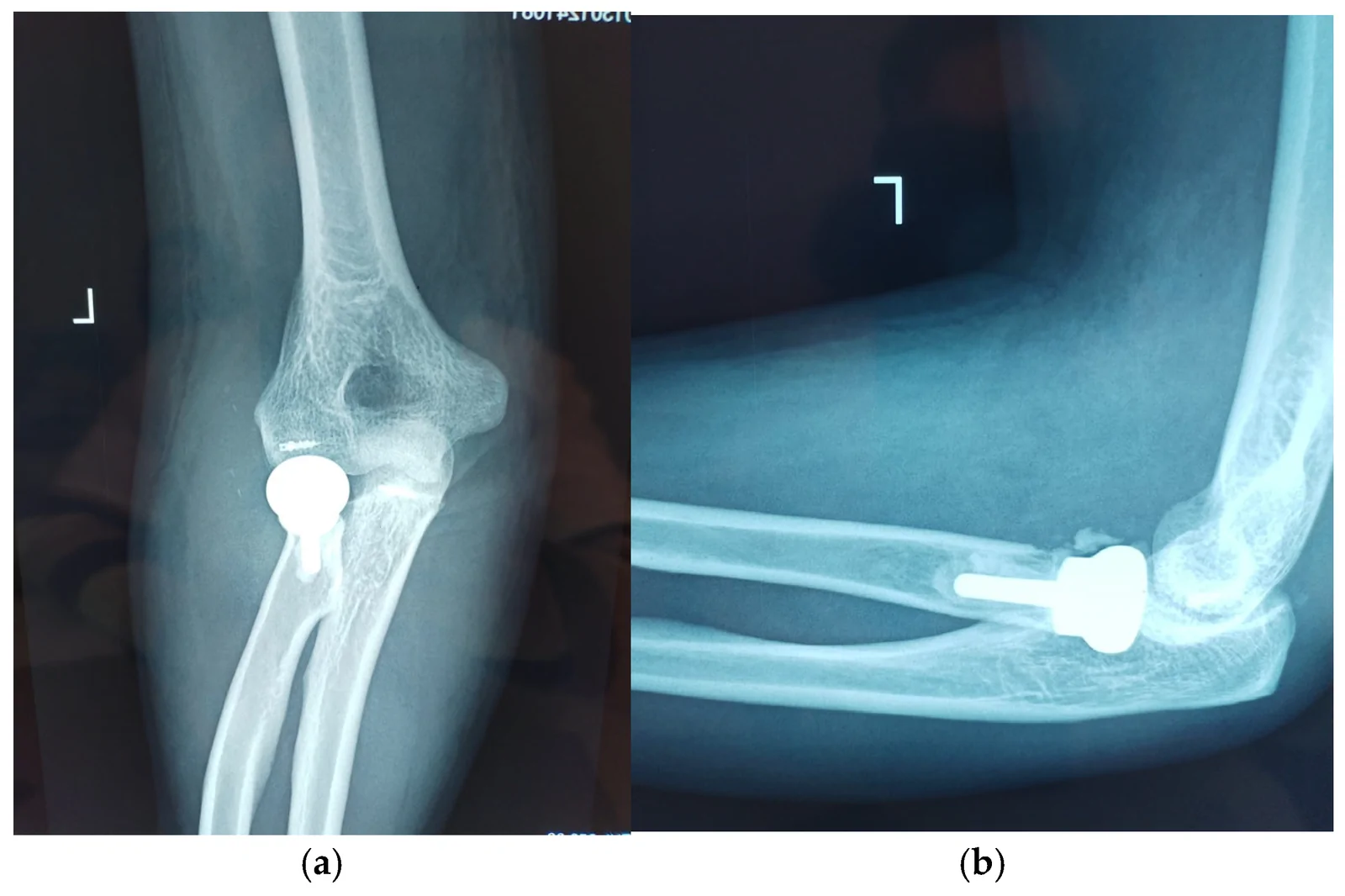
(**a**,**b**) Radial head arthroplasty was performed using the ExploR^®^ Modular Radial Head System prosthesis (Zimmer Biomet).

Following capsular exposure, contracted capsular structures contributing to stiffness were systematically released. Osteophytes, loose bodies, and radiocapitellar impingement were addressed as indicated to restore motion. Both anterior and posterior capsular releases were performed through the same approach in order to achieve satisfactory improvement in the range of motion. In two cases, radial head arthroplasty was performed using the ExploR^®^ Modular Radial Head System prosthesis (Zimmer Biomet, Zug, Switzerland) (Figure 3).

The main advantages of this approach included preservation of the extensor mechanism, limited soft-tissue dissection, reduced wound morbidity, and the ability to initiate early postoperative rehabilitation. This approach proved particularly useful in patients with predominantly lateral pathology and in cases that did not require ulnar nerve decompression or medial-sided reconstructive procedures [19].

When the isolated lateral approach was insufficient to completely expose the elbow joint, a combined medial approach was utilized. This decision was made to preserve the integrity of the triceps tendon, thereby avoiding secondary dysfunctions that may occur after its detachment and subsequent reconstruction.

The medial approach provided direct access to the anterior and posterior aspects of the ulno-humeral joint while allowing identification and protection of the ulnar nerve throughout the procedure. All procedures were performed by the senior author. A longitudinal incision centered over the medial epicondyle was utilized, and the ulnar nerve was routinely identified, carefully released, and protected during the operation. An anterior subcutaneous transposition of the ulnar nerve was systematically performed in all cases at the end of surgery to minimize postoperative nerve irritation and to facilitate the extensive soft-tissue releases required for restoration of elbow motion.

Following exposure, the flexor-pronator mass was elevated as required to gain access to the joint capsule. Contracted anterior and posterior capsular structures contributing to stiffness were systematically released. Osteophytes involving the coronoid process, coronoid fossa, olecranon, and olecranon fossa were excised when present, and loose bodies were removed to eliminate mechanical blocks to motion. Particular attention was paid to restoring ulno-humeral congruity while preserving the integrity and stability of the medial collateral ligament complex. When gross medial instability or structural incompetence was identified, reconstruction of the medial collateral ligament was performed using SwiveLock^®^ anchors and FiberWire^®^ sutures (Arthrex).

The main advantages of the medial approach included excellent visualization of the ulno-humeral articulation, reliable identification and management of the ulnar nerve, and effective treatment of pathology involving the medial compartment of the elbow. This approach proved particularly useful in patients with severe flexion contractures requiring extensive anterior release and in cases in which concomitant ulnar nerve decompression and anterior transposition were indicated [20].

**Figure 4:**
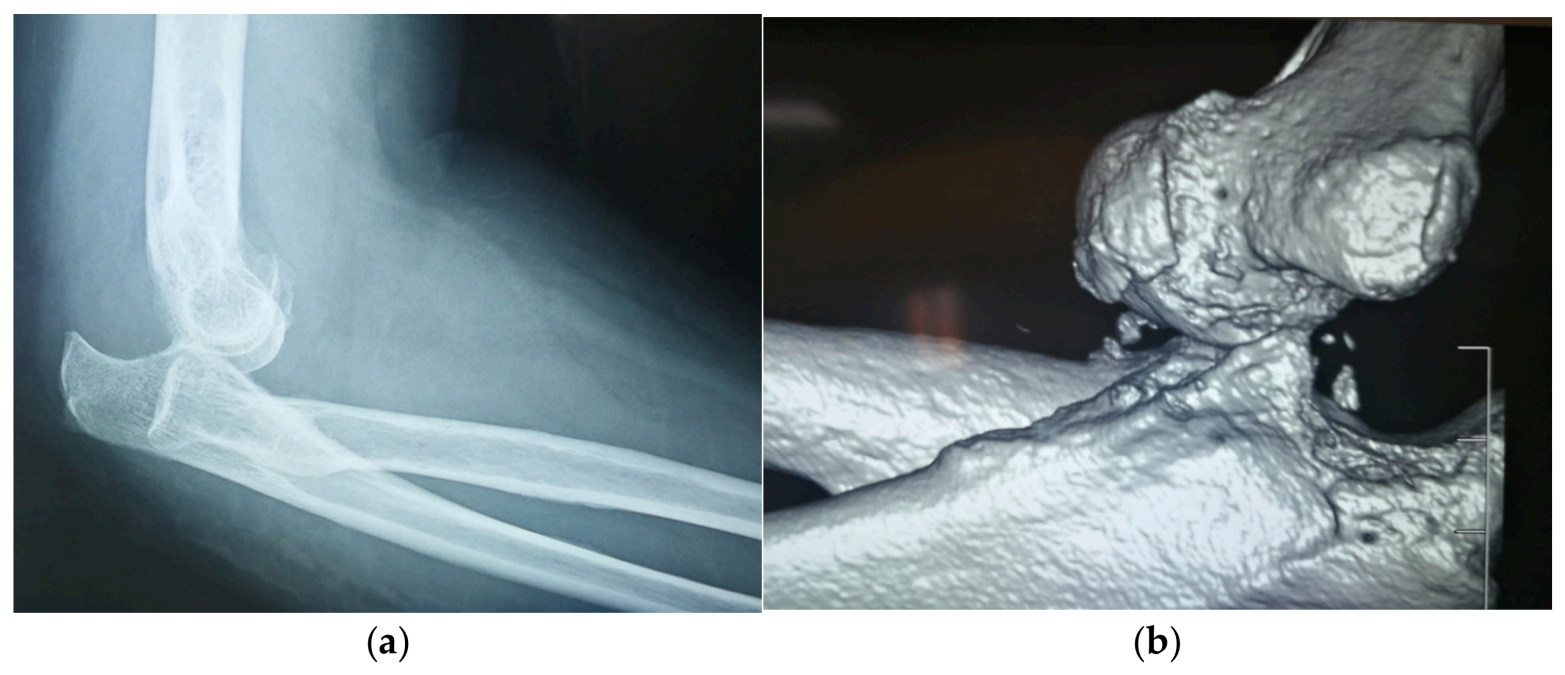

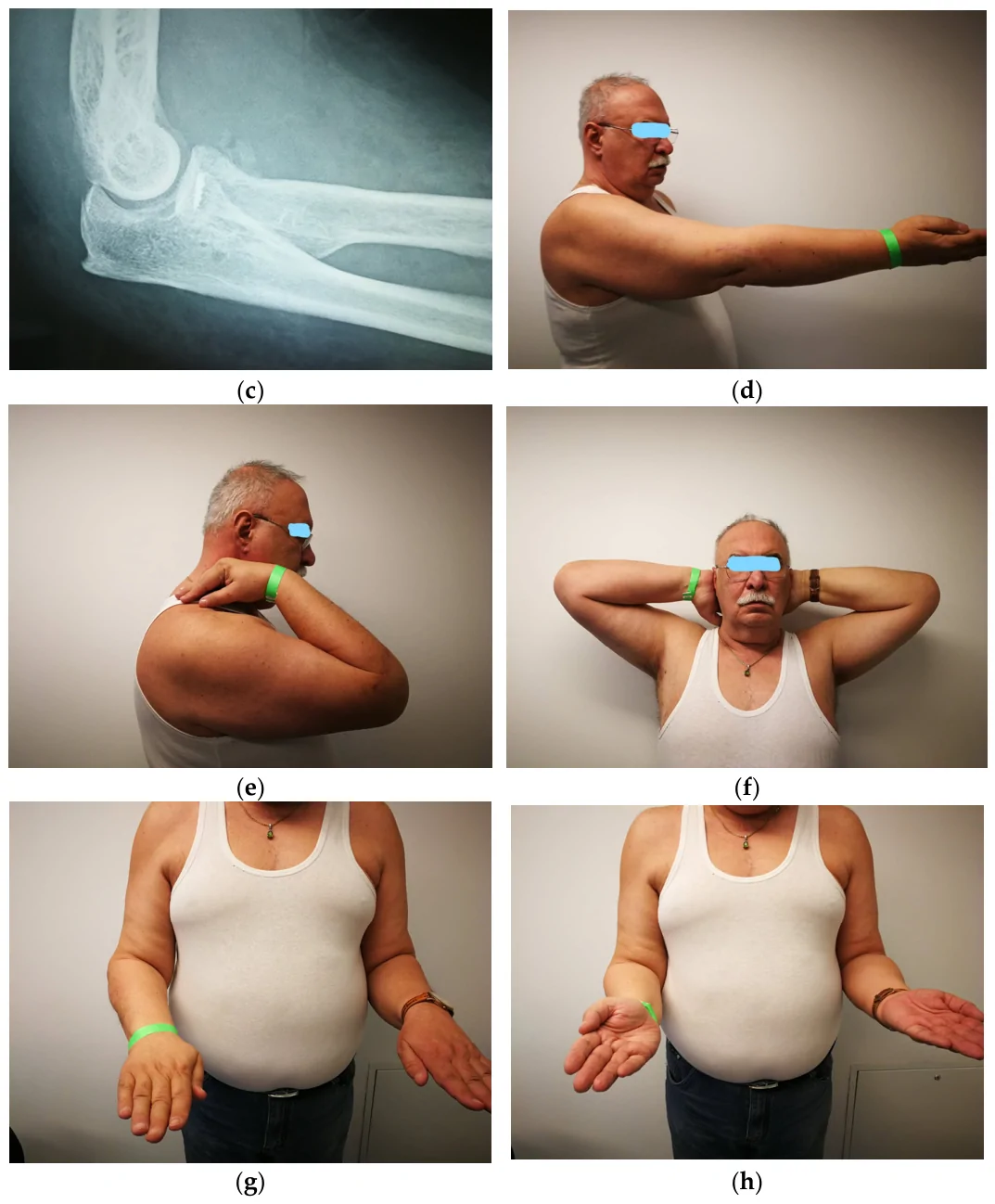
(**a**–**c**) Old neglected posterior elbow posterior luxation treated by open reduction, anterior capsulorrhaphy with a Fastak anchor, and lateral collateral ligament reconstruction using a gracilis autograft and SwiveLock anchors. (**d**–**h**) Postoperative aspect at one year (lateral approach).

In four patients with a coronoid process defect and posterior subluxation, the anterior capsule was reattached to the remaining coronoid stump using Fastack anchors (Arthrex) (Figure 4).

**Figure 5:**
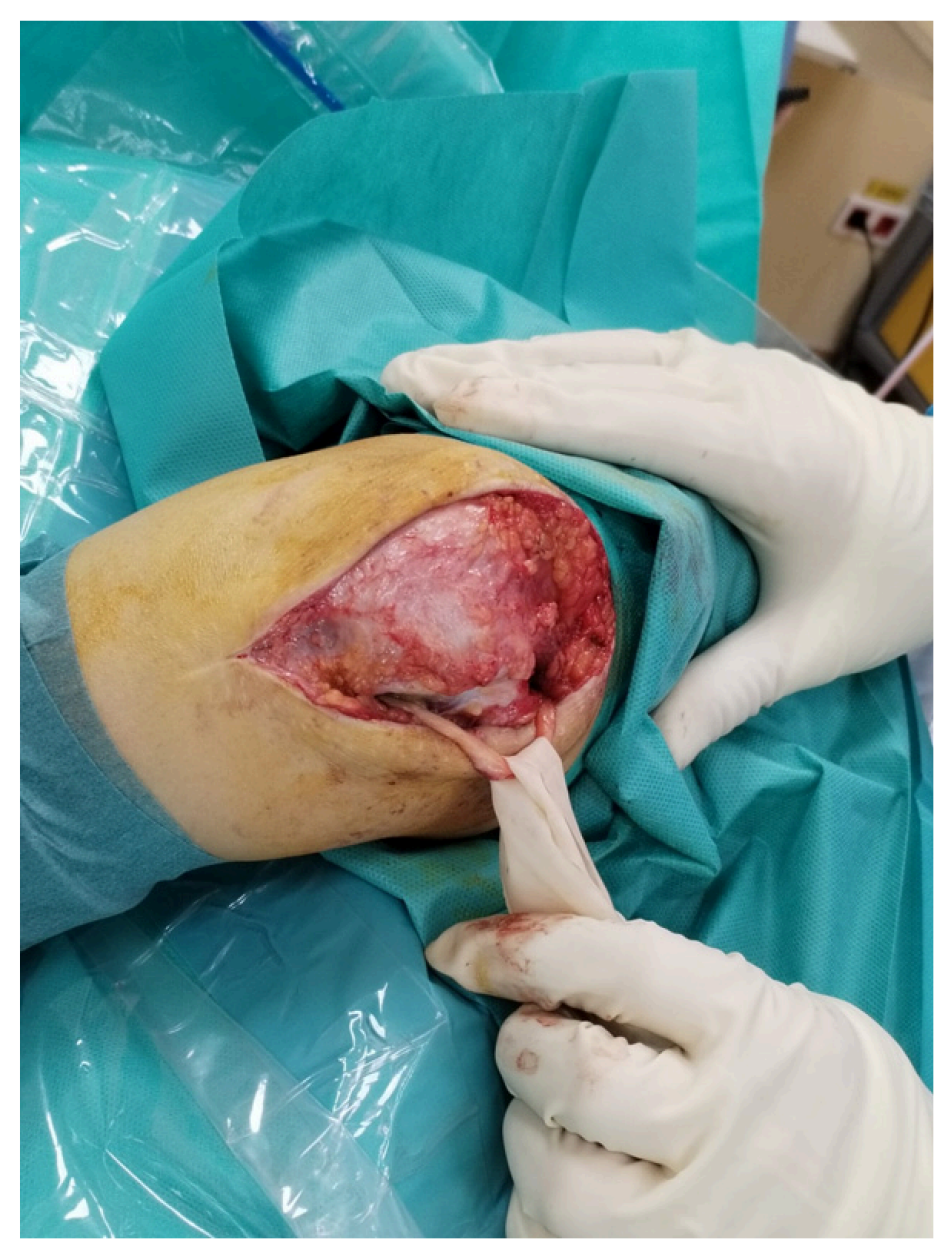
Posterior approach with ulnar nerve isolation.

The posterior approach to the elbow was preferred in cases offering highly complex, multi-column global stiffness, as it ensured optimal visualization and access for the planned surgical procedure. This approach was chosen not only for its wide operative field but also for its versatility in managing complex intra-articular and periarticular pathology [8] (Figure 5).

A key advantage in these cases was the ability to perform simultaneous triceps tendon lengthening using a V-shaped tricepsplasty. This technique facilitated controlled mobilization of the extensor mechanism, allowing adequate joint exposure without necessitating complete detachment of the triceps insertion. As a result, it helped preserve the continuity and function of the extensor apparatus while minimizing the risk of postoperative weakness or dysfunction.

Overall, the posterior approach combined with V-shaped tricepsplasty provided a reliable balance between exposure and soft-tissue preservation, making it particularly suitable in cases requiring both complex access and careful protection of the triceps mechanism [21].

### 2.5. Postoperative Rehabilitation and Follow-Up

Early mobilization was initiated in all patients as soon as clinically tolerated, under supervision of a physiotherapist, knowing that the maximum range of motion is achieved during the first postoperative weeks [5,22,23,24]. The goal of postoperative rehabilitation is to maintain the maximum ROM gained during surgery. Adjuvant splinting can be used effectively to treat contractures resistant to standard exercise programs. Rehabilitation protocols were individualized depending on the extent of soft-tissue release and associated reconstructive procedures [25,26]. The program emphasized progressive restoration of flexion–extension and forearm rotation while protecting repaired structures when applicable.

In patients who underwent ligament reconstruction, flexion–extension mobilization during the first six weeks was performed exclusively in the sagittal plane, with varus and valgus stresses being avoided. In patients who underwent triceps lengthening, emphasis during the first six weeks was placed on passive flexion–extension exercises, while excessive flexion and overloading of the repair were avoided.

Follow-up evaluations were systematically performed at 2 weeks, 6 weeks, 3 months, 6 months, and 12 months postoperatively. The 12-month interval was strictly utilized as the definitive, uniform endpoint for all 20 consecutive cases to ensure statistical homogeneity during pre- and postoperative comparisons. For a subset of patients, extended clinical surveillance was maintained for up to 2 to 3 years to monitor long-term joint behavior.

### 2.6. Statistical Analysis

Continuous variables, including range of motion parameters, were expressed as means ± standard deviations (SD). The statistical significance of preoperative to postoperative improvements was evaluated using the paired *t*-test. To assess the clinical magnitude of the treatment effect, Cohen’s *d* effect sizes were calculated using standard pooled standard deviation formulas for paired samples.

The resulting effect sizes were interpreted according to Cohen’s thresholds: 0.20 for a small effect, 0.50 for a medium effect, and 0.80 for a large effect, with values exceeding 2.0 considered indicative of an extremely large clinical impact. All mathematical computations were processed using standard digital statistical calculators.

## 3. Results

### 3.1. Patient Demographics and Baseline Characteristics

A total of 20 consecutive patients underwent surgical treatment for post-traumatic elbow stiffness and completed a minimum follow-up of 12 months. The cohort consisted of 14 males (70%) and 6 females (30%), with a mean age of 43.8 years (range, 24–66 years) (Table 2).

The etiologies of elbow stiffness included distal humeral fractures (45%), terrible triad injuries (20%), elbow dislocations (15%), olecranon fractures (10%), and neglected radial head fractures (10%) (Table 3).

All patients had failed nonoperative treatment, including physiotherapy, and presented with substantial functional limitations affecting activities of daily living.

### 3.2. Range of Motion Outcomes

**Figure 6:**
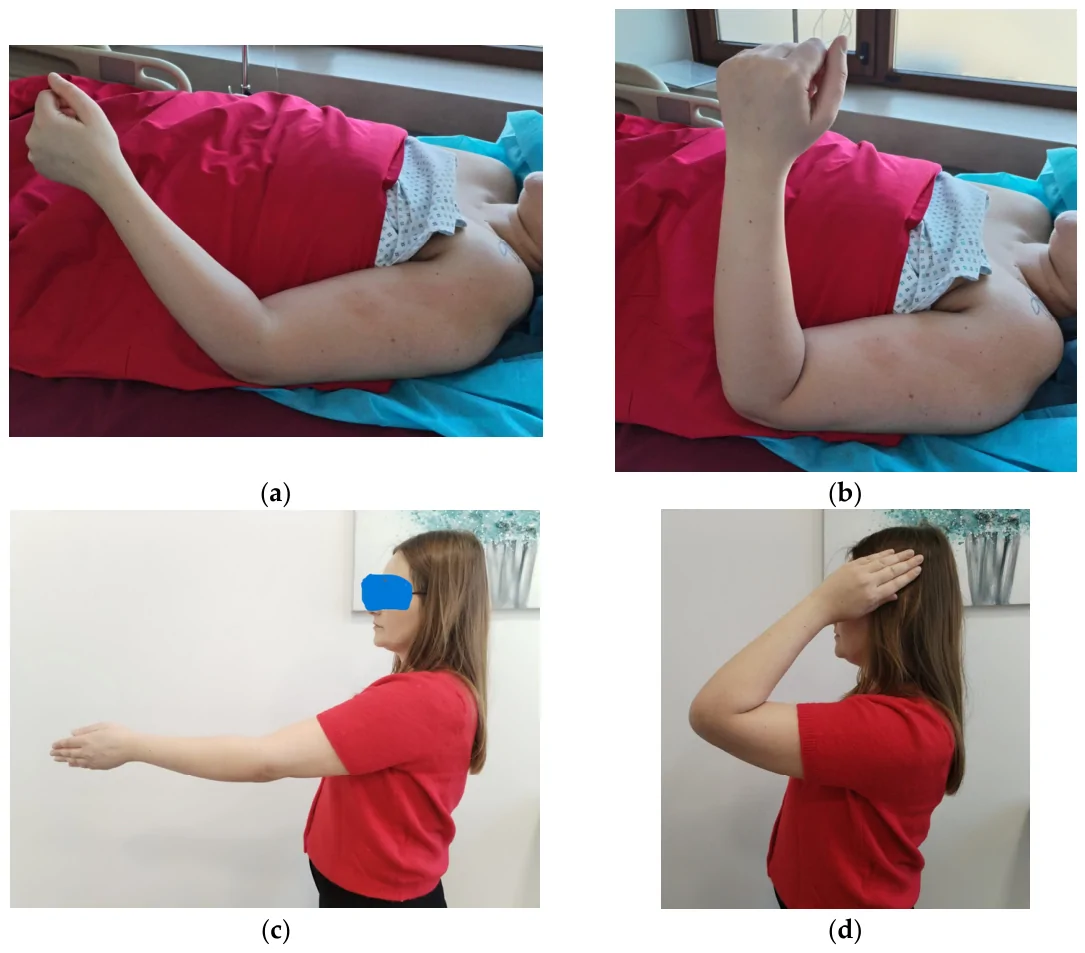

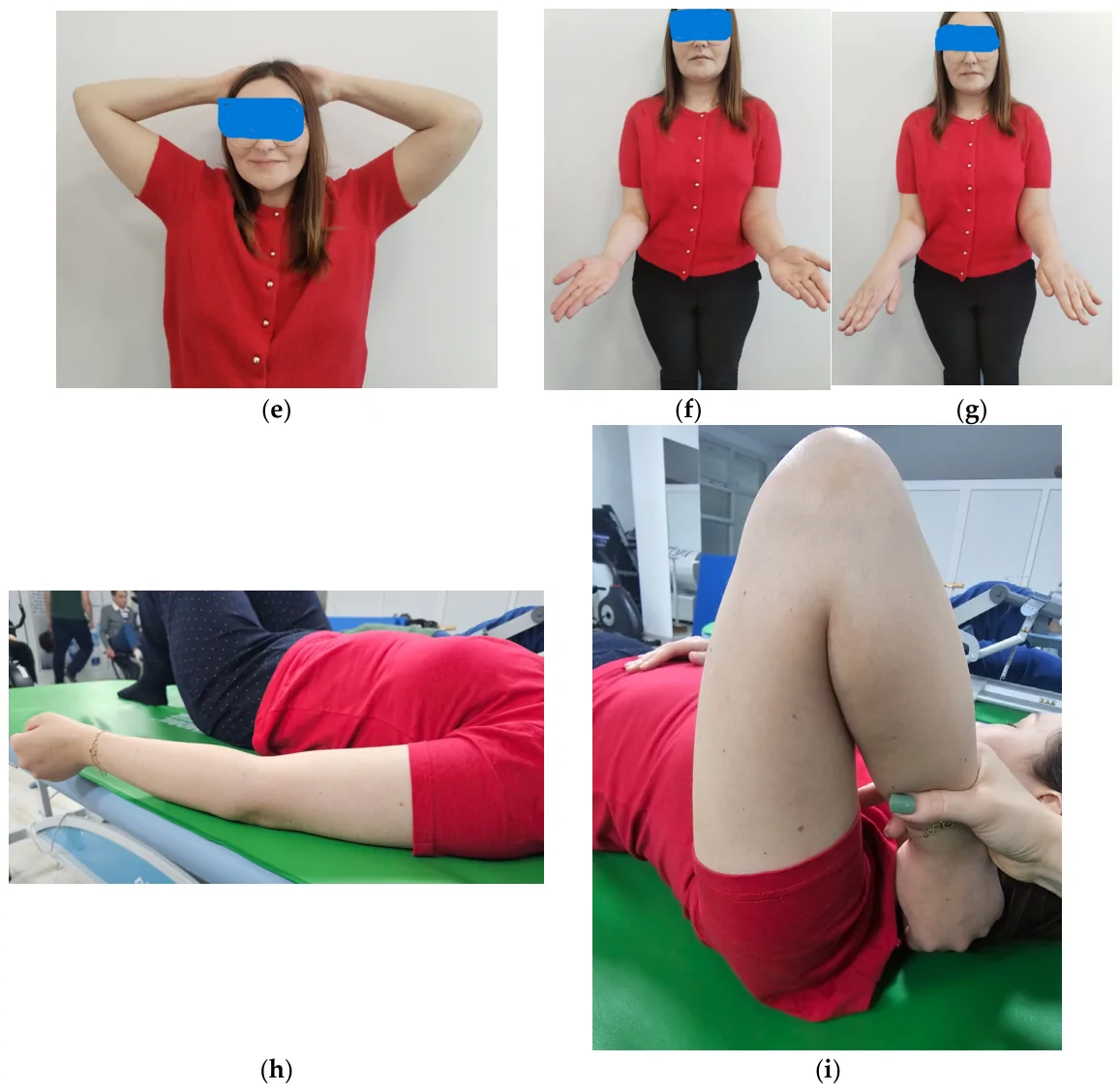
Secondary elbow stiffness after posterior elbow dislocation treated conservatively. (**a**,**b**) Preoperative flexion–extension arc: 70–90°. (**c**–**g**) Postoperative outcome at 6 weeks and (**h**,**i**) 3 months (posterior approach).

Significant improvements were observed in all measured parameters of elbow and forearm motion following surgical treatment (Figure 6).

Mean elbow flexion improved from 81.0 ± 14.8° preoperatively to 125.3 ± 9.8° postoperatively (*p* < 0.0001).

The mean extension deficit improved from 66.5 ± 19.3° to 16.5 ± 13.9° (*p* < 0.0001).

Forearm supination improved from 54.8 ± 42.5° to 82.8 ± 18.2° (*p* = 0.0028), while pronation improved from 67.0 ± 29.5° to 84.5 ± 11.2° (*p* = 0.0019) (Table 4).

The overall flexion–extension arc increased from **14.5 ± 18.8°** preoperatively to **108.8 ± 22.5°** postoperatively, representing a mean gain of **94.3°** (*p* < 0.001).

Most patients achieved a final functional arc exceeding 100°, allowing performance of the majority of activities of daily living.

### 3.3. Functional Outcomes

Substantial functional improvement was observed after surgery.

The mean Mayo Elbow Performance Score (MEPS) improved from **30.0 ± 7.6** preoperatively to **93.3 ± 6.1** postoperatively, corresponding to an improvement from a poor to an excellent functional category.

Similarly, the mean Disabilities of the Arm, Shoulder and Hand (DASH) score improved from **92.9 ± 10.3** preoperatively to **18.8 ± 13.4** postoperatively, indicating a marked reduction in upper-extremity disability (Table 5).

The mean postoperative Visual Analog Scale (VAS) score was **0.45 ± 0.69**, reflecting minimal residual pain at final follow-up.

### 3.4. Effect Size Analysis

Continuous variables, including range of motion parameters, were expressed as means ± standard deviations (SD). The statistical significance of preoperative to postoperative improvements was evaluated using the paired *t*-test. To assess the clinical magnitude of the treatment effect, Cohen’s *d* effect sizes were calculated using standard pooled standard deviation formulas for paired samples. The resulting effect sizes were interpreted according to Cohen’s thresholds: 0.20 for a small effect, 0.50 for a medium effect, and 0.80 for a large effect, with values exceeding 2.0 considered indicative of an extremely large clinical impact. All mathematical computations were processed using standard digital statistical calculators.

Effect size analysis demonstrated substantial clinical improvement across all measured parameters. The largest treatment effect was observed for the flexion–extension arc (Cohen’s d: 4.55), followed by elbow flexion (d = 3.53) and correction of extension deficit (d = 2.97). Improvements in forearm supination (d = 0.86) and pronation (d = 0.78) corresponded to large effect sizes (Table 6).

These findings indicate that the observed improvements were not only statistically significant but also clinically meaningful.

### 3.5. Surgical Procedures

All patients underwent anterior arthrolysis, posterior arthrolysis, and triceps tenolysis.

Additional procedures were performed according to the underlying pathology:

- Ulnar nerve transposition: 12 patients (60%);
- Tricepsplasty: 8 patients (40%);
- Combined medial and lateral collateral ligament reconstruction: 4 patients (20%);
- Isolated lateral collateral ligament reconstruction: 2 patients (10%);
- Anterior capsule repair/reinsertion: 4 patients (20%);
- Radial head arthroplasty: 2 patients (10%) (Table 7).

### 3.6. Complications

Three complications were recorded during follow-up, corresponding to an overall complication rate of **15%**. One patient developed postoperative sensory ulnar neuropathy in the setting of extensive heterotopic ossification surrounding the ulnar nerve. Symptoms gradually improved during follow-up without additional surgery. One patient developed postoperative triceps insufficiency following tricepsplasty and required revision surgery with triceps reinsertion. Final functional recovery was satisfactory.

In the remaining eight patients who underwent tricepsplasty, only mild triceps weakness was observed. However, this did not impair normal elbow function and did not require revision or additional reconstructive surgery.

**Figure 7:**
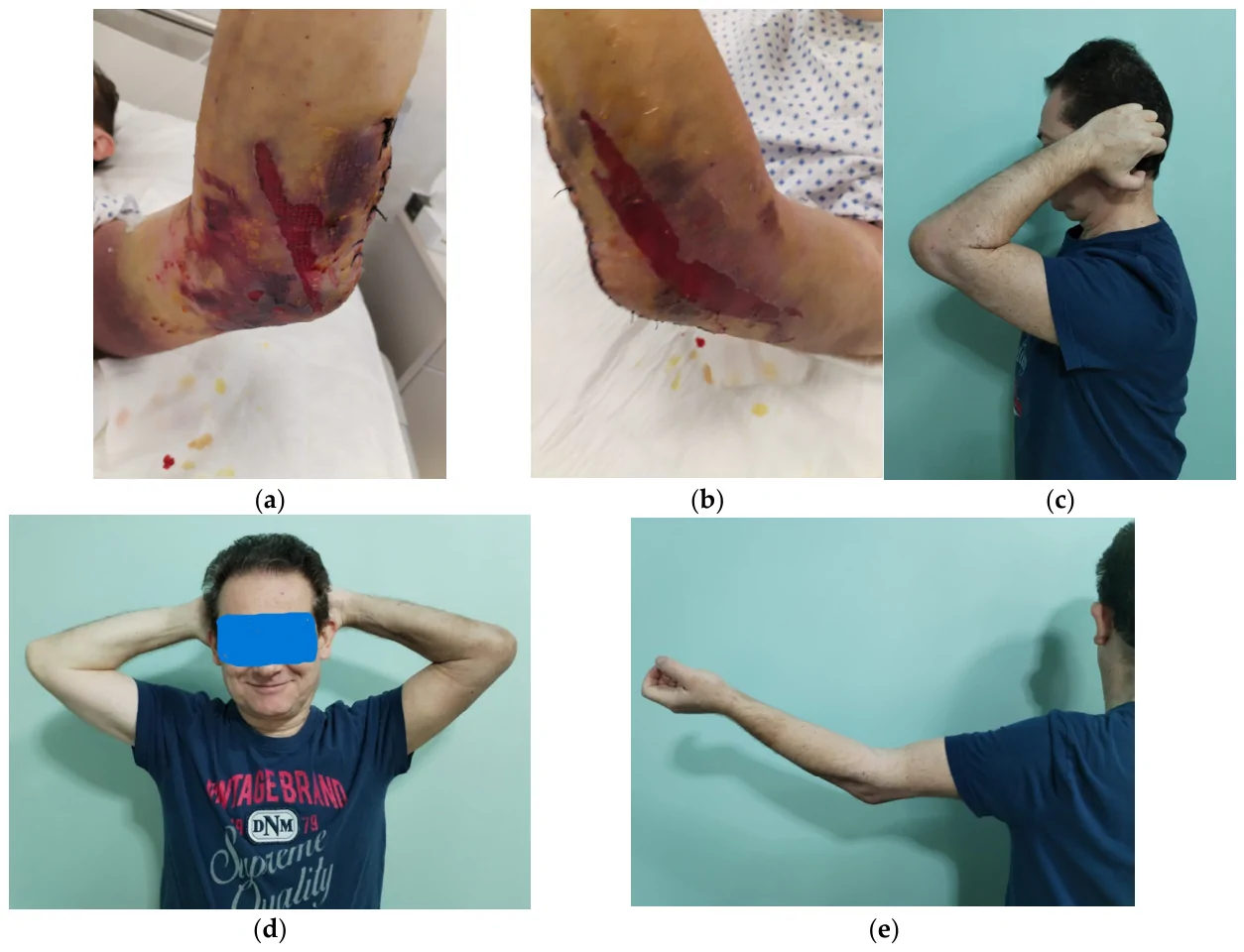
(**a**,**b**) Skin blistering following a posterior approach at a patient with history of systemic vasculitis (medial and lateral sides). (**c**–**e**) At 6 weeks postoperative view.

One patient developed postoperative skin blistering following a posterior approach. This patient had a known history of systemic vasculitis and previous wound-healing complications after other surgical procedures (Figure 7). The skin lesions resolved with local wound care and observation without infection or further intervention (Table 8).

No deep infections, recurrent instability, implant failure, or recurrent stiffness requiring reoperation were observed.

To ensure maximum transparency regarding the tailored treatment approach, a patient-specific correlation matrix was developed. The distribution and severity of capsular contracture, heterotopic ossification (HO), articular incongruity, and periarticular soft-tissue fibrosis across individual patients, along with their corresponding combinations of adjunctive surgical procedures, are comprehensively detailed in Table 9. This matrix highlights how additional procedures were customized at the patient level to address specific secondary pathologies.

## 4. Discussion

### 4.1. Principal Findings

The optimal timing of surgical intervention for post-traumatic elbow stiffness remains controversial. While some authors advocate delaying surgery until approximately 12 months after injury to reduce the risk of secondary inflammation that may adversely affect postoperative outcomes [16], others support earlier intervention, typically between 6 and 8 months after trauma, having found no significant differences in postoperative range of motion, functional outcomes, or complication rates between early and delayed treatment groups [17]. For this reason, in our series, all patients underwent surgery between 6 and 12 months after the initial traumatic event.

The main finding of this study is that open arthrolysis, combined with pathology-specific adjunctive procedures, led to significant and clinically relevant improvements in elbow mobility and function in patients with post-traumatic elbow stiffness. The advantage of open arthrolysis compared to other techniques, such as arthroscopy, is that it allows comprehensive access to both lateral and medial structures, with the possibility of ligament reconstruction when required [16]. In addition, the posterior approach enables triceps tendon lengthening, which contributes to a significant improvement in postoperative flexion.

The mean gain of 94.3° in the flexion–extension arc is particularly notable, demonstrating a substantial recovery of elbow mobility. Significant improvements in forearm pronation and supination were also observed, highlighting the effectiveness of a thorough and complete surgical release in restoring multi-planar forearm function.

The increase in MEPS from 30.0 to 93.3 points, alongside the reduction in DASH from 92.9 to 18.8 points, demonstrates that the gains in range of motion translated into meaningful improvements in daily function and overall quality of life.

### 4.2. Comparison with Previous Literature

The treatment of post-traumatic elbow stiffness continues to be a demanding endeavor for upper-limb surgeons. Previous studies have demonstrated that successful treatment requires identification and correction of all contributing factors, including capsular fibrosis, heterotopic ossification, osseous impingement, muscle contracture, and residual instability [24,27,28,29]. The diverse etiology of our 20-case series, ranging from complex distal humerus fractures (45%) to terrible triads (20%) and neglected radial head fractures (10%), underscores the multifactorial nature of arthrofibrosis development, as extensively mapped in bioinformatic and critical analyses by Morrey et al. [27] and Attum & Obremskey [28].

The cornerstone of achieving a functional range of motion (ROM) in these rigid joints is a comprehensive and structured surgical release. Our patients achieved an outstanding average gain in the total arc of flexion–extension of 94.3° (moving from a severely restricted 14.5° preoperatively to a functional 108.8° postoperatively). This massive gain likely reflects the comprehensive approach employed, including routine anterior and posterior arthrolysis, triceps tenolysis, and selective adjunctive reconstructive procedures.

When comparing our kinematics data to contemporary literature, our results align closely with the recent systematic review by Fabian et al., which validated the high success rates of open arthrolysis in large-scale data [16]. Furthermore, our approach to complex cases requiring associated reconstructions proved highly effective. For instance, our subset of patients who underwent radial head arthroplasty (*n* = 2) or collateral ligament reconstructions (*n* = 6) achieved stable, mobile joints without recurrent instability. This is consistent with the findings of Gang et al., who demonstrated that combining open arthrolysis with radial head arthroplasty yields durable, long-term results exceeding 8 years [30]. It also echoes the work of Marco et al. [31] regarding the necessity of restoring ligamentous stability (such as LUCL reconstruction) to secure long-term mobility and prevent failure.

Surgical approach and patient selection are critical modulators of the final outcome. In our cohort, the posterior approach was favored in 45% of cases, followed by the lateral (35%) and combined lateral-medial approach (20%). The choice of these exposures allowed excellent access to both the anterior and posterior compartments, minimizing the risk of incomplete release. This tactical customization is supported by Nikku et al., who emphasized that the surgical approach and strict adherence to mobilization protocols are paramount preoperative and intraoperative predictors of success [32].

Ultimately, the ultimate measure of surgical success lies in functional restoration and patient-reported outcomes. The postoperative MEPS of 93.3 points achieved in our cohort is fully comparable to the excellent outcomes reported in contemporary elbow arthrolysis literature [16,30,31,32,33,34], shifting our patients’ status from “Poor” to “Excellent”. This clinical success is further objectified by the drastic drop in the DASH score (from 93.0 to 18.8) and the near-complete eradication of pain, with the VAS score decreasing from 7.82 to 0.45. Similar long-term functional turnarounds and pain relief profiles after open contracture release were documented by Sun et al. [33] and in the long-term case series by Brittany et al.

The low rate of major complications in our series, with only one case of secondary triceps insufficiency following tricepsplasty, further reinforces the safety profile of our routine open release protocol when compared to the broader complication rates sometimes reported in historical cohorts.

### 4.3. Importance of Stability Restoration

Residual instability frequently contributes to both pain and recurrent stiffness, creating a paradoxical clinical challenge where a loose joint becomes fibrotic due to chronic inflammation and protective muscle guarding. In our series, ligament reconstruction was required in six patients (four combined LCL and MCL reconstructions, and two isolated LCL reconstructions) [35,36].

Failure to address instability at the time of arthrolysis may significantly compromise long-term outcomes and lead to early failure. Recent literature strongly reinforces this dual approach. Marco et al. [31] demonstrated excellent minimum 2-year outcomes when performing lateral ulnar collateral ligament (LUCL) reconstructions to address posterolateral rotatory instability, emphasizing that structural competence prevents mechanical wear and recurrent contracture. This paradigm is further supported by a comprehensive clinical review by Christopher et al. [37], which proved that ligament repairs and reconstructions in chronic instability settings not only effectively stabilize the joint but also directly yield significant improvements in the overall postoperative range of motion (ROM) [38].

Furthermore, in complex cases involving severe post-traumatic pathology or chronic dislocations, recent evidence by Anna E et al. [39] emphasizes that restoring normal anatomical relationships and reconstructing collateral ligament stability is just as critical as the open release itself to ensure stable functional recovery. More recently, innovative approaches by Mehmet F. et al. [40] regarding total collateral ligament reconstruction during stiff elbow release highlight that addressing ligamentous insufficiency is a viable, reliable, and mandatory solution to sustain the achieved surgical arc of motion. Consequently, our results strongly support the concept that restoration of elbow stability should be considered an essential, non-negotiable component of surgical management whenever ligament insufficiency is intraoperatively identified.

### 4.4. Ulnar Nerve Management

The ulnar nerve represents a particular concern during extensive elbow release procedures, as the acute increase in postoperative flexion places the nerve under sudden mechanical elongation. In our series, ulnar nerve transposition was performed in 60% of patients (12 out of 20 cases), resulting in only one transient postoperative neuropathy that fully resolved during follow-up. This excellent safety profile was maintained despite the high prevalence of severe contractures and heterotopic ossification within our cohort. These findings align with a recent comparative study by Jiajun X et al., which demonstrated that anterior transposition of the ulnar nerve yields vastly superior neurological outcomes and minimizes delayed traction neuritis in patients presenting with severe mechanical blocks or preoperative symptoms [41].

### 4.5. Open Versus Arthroscopic Release in Stiff Elbow

Although several authors have reported favorable outcomes following arthroscopic joint release for the treatment of elbow stiffness [42,43,44,45], our institutional experience with arthroscopic elbow procedures for this specific condition remains limited. Arthroscopic release has shown good mid-term results in milder extrinsic contractures. However, it presents inherent limitations in cases of severe stiffness accompanied by structural joint deformities or when heterotopic ossification carries a risk of neurovascular injury. Open surgery remains the preferred and safest modality for patients requiring concurrent complex procedures, including ulnar nerve transposition, triceps tendon lengthening, hardware removal, or collateral ligament reconstruction.

### 4.6. Functional Recovery and Complications

The dramatic improvement in both MEPS and DASH scores observed in this study indicates that gains in motion translated into meaningful improvements in daily activities, achieved without persistent pain as reflected by the very low postoperative VAS scores. Our overall complication rate of 15% is comparable to rates reported in previous studies of open elbow arthrolysis. Importantly, only one patient required revision surgery. The postoperative skin complication occurred in a patient with pre-existing systemic vasculitis and a known history of wound-healing difficulties, suggesting that this event was primarily related to patient-specific biological factors rather than surgical technique. No deep infections, recurrent instability, implant failures, or recurrent stiffness requiring reoperation were observed.

### 4.7. Limitations

This study has several limitations that should be acknowledged. First, its retrospective design introduces the potential for selection bias. Second, the sample size is relatively small (*N* = 20), reflecting the strict inclusion criteria and the specific nature of complex open arthrolysis performed at a single tertiary orthopedic center. Third, the study population demonstrated considerable heterogeneity in initial injury patterns (ranging from complex distal humeral fractures to neglected dislocations) and the subsequent combination of adjunctive surgical procedures. Because several etiological subgroups were represented by only two or three patients, meaningful subgroup analyses or direct intergroup statistical comparisons were precluded. Consequently, the sample size within each etiology subgroup remains insufficient to support robust statistical conclusions regarding whether specific injury mechanisms directly alter chronological kinematic recovery or final functional outcomes.

Furthermore, although a detailed description of the core surgical technique is provided, the study population is limited by the lack of quantified preoperative grading regarding the exact distribution and severity of capsular contracture, heterotopic ossification, and articular incongruity for each isolated patient level. Lastly, regarding the follow-up period, clinical evaluations were strictly standardized at fixed intervals (2 weeks, 6 weeks, 3 months, 6 months, and 12 months) to maintain chronological consistency and safeguard statistical homogeneity across the entire cohort for pre- and postoperative comparisons. For a subset of patients, extended clinical surveillance was maintained for up to 2 to 3 years to monitor long-term joint behavior. While a 12-month endpoint provides valuable immediate insights into early functional gains, it is relatively short to evaluate the long-term durability of the surgical release. However, clinical evidence suggests that if satisfactory functional range and joint stability are preserved at 1 year postoperatively, the risk of secondary regression decreases significantly as capsular tissue remodeling stabilizes [8]. We think that a longer standardized follow-up is critical to definitively monitor for late complications, such as the gradual recurrence of stiffness, progressive heterotopic ossification, delayed instability, or the eventual need for secondary procedures. Nevertheless, prospective, multicenter studies with larger, etiology-specific cohorts and longer standardized follow-up tracking remain necessary to definitively map the long-term outcomes of this comprehensive surgical approach.

## 5. Conclusions

Open arthrolysis combined with pathology-specific adjunctive procedures is a reliable and effective treatment for post-traumatic elbow stiffness.

The procedure resulted in significant improvements in elbow motion, forearm rotation, pain, and upper-extremity function. The mean flexion–extension arc improved by 94.3°, while MEPS increased from 30.0 to 93.3 points and DASH decreased from 92.9 to 18.8 points.

The large effect sizes observed across all motion parameters confirm that the improvements were not only statistically significant but also clinically meaningful.

When combined with appropriate rehabilitation and treatment of associated pathology, open arthrolysis can restore a functional range of motion and achieve excellent functional outcomes with an acceptable complication profile.

## Figures and Tables

**Table 1:** Selection of Surgical Approach.

Lateral Approach Focus	Combined Medial-Lateral Approach	Posterior Approach Focus
Extrinsic/combined contractures	Severe flexion contractures Extensive anterior capsule resection needed	Highly complex, multi-column global stiffness Severe distal humeral malunions
Predominantly anterolateral pathology	Medial osseous impingement/HO	Preoperative triceps shortening
Preserved triceps mechanism	Coronoid osteophytes/ Ulnar compression Triceps tendon integrity preserved	Circumferential exposure of both columns required
Absence of ulnar nerve symptoms Indicated if varus laxity requires LCL repair	Indicated if varus/valgus laxity requires LCL and MCL repair	

LCL: lateral collateral ligament. MCL: medial collateral ligament. HO: heterotopic ossification.

**Table 2:** Patient Demographics.

Variable	Value
Number of patients	20
Mean age (years)	43.8 ± 11.6
Male	14 (70%)
Female	6 (30%)

**Table 3:** Etiology of stiffness.

Cause	*n* (%)
Distal humeral fracture	9 (45%)
Terrible triad injury	4 (20%)
Elbow dislocation	3 (15%)
Olecranon fracture	2 (10%)
Radial head fracture	2 (10%)

**Table 4:** Range of Motion Outcomes.

Parameter	Preoperative (Mean ± SD)	Postoperative (Mean ± SD)	Improvement	*p*-Value
Flexion (°)	81.0 ± 14.8	125.3 ± 9.8	+44.3	<0.001
Extension deficit (°)	66.5 ± 19.3	16.5 ± 13.9	−50.0	<0.001
Flexion–extension arc (°)	14.5 ± 18.8	108.8 ± 22.5	+94.3	<0.001
Supination (°)	54.8 ± 42.5	82.8 ± 18.2	+28.0	0.003
Pronation (°)	67.0 ± 29.5	84.5 ± 11.2	+17.5	0.002

**Table 5:** Functional Outcomes.

Outcome Measure	Preoperative (Mean ± SD)	Postoperative (Mean ± SD)
MEPS	30.0 ± 7.6	93.3 ± 6.1
DASH	92.9 ± 10.3	18.8 ± 13.4
VAS	7.82 ± 0.94	0.45 ± 0.69

MEPS: Mayo Elbow Performance Score. DASH: Disabilities of the Arm, Shoulder and Hand. VAS: Visual Analog Scale.

**Table 6:** Effect Size Analysis.

Parameter	Cohen’s d	Interpretation
Flexion	3.53	Extremely large
Extension deficit	2.97	Extremely large
Flexion–extension arc	4.55	Extremely large
Supination	0.86	Large
Pronation	0.78	Large

**Table 7:** Surgical Procedures.

Procedure	*n* (%)
Ulnar nerve transposition	12 (60%)
Tricepsplasty	8 (40%)
Combined MCL + LCL reconstruction	4 (20%)
Isolated LCL reconstruction	2 (10%)
Anterior capsule repair	4 (20%)
Radial head arthroplasty	2 (10%)

MCL: Medial collateral ligament. LCL: Lateral collateral ligament.

**Table 8:** Complications.

Complication	*n* (%)
Sensory ulnar neuropathy	1 (5%)
Triceps insufficiency requiring revision	1 (5%)
Skin blistering in patient with vasculitis	1 (5%)
Total complications	3 (15%)

**Table 9:** Patient-specific distribution of baseline etiologies, findings, and adjunctive procedures (*N* = 20) *.

Patient	Etiology	Soft tissue Pathology & Severity	Bony Alterations & Incongruity	Adjunctive Surgical Procedures
P1	Distal humeral Fr	Severe ant/post capsular fibrosis	Olecranon fossa osteophytes	V-tricepsplasty, UN transposition
P2	Distal Humeral Fr	Severe global fibrosis, triceps adhesion	Coronoid osteophytes hardware	V-tricepsplasty, UN transposition, HW removal
P3	Distal Humeral Fr	Moderate ant capsular contracture	HO class I	UN transposition
P4	Distal Humeral Fr	Severe post capsular Contracture	Olecranon fossa osteophytes	V-tricepsplasty, UN osteophyte excision
P5	Distal Humeral Fr	Moderate global periarticular fibrosis	Retained hardware, no HO	HW removal
P6	Distal Humeral Fr	Severe ant contracture, triceps shortening	HO Class II minor incongruity	V-tricepsplasty, UN transposition
P7	Distal Humeral Fr	Severe post capsular contracture	Olecranon/coronoid osteophytes	V-tricepsplasty, UN transposition
P8	Distal Humeral Fr	Moderate ant capsular contracture	HO Class I	UN transposition
P9	Distal Humeral Fr	Moderate post-periarticular fibrosis	Minor osteophytes, no HO	UN transposition
P10	Terrible Triad	Severe global fibrosis LCL/MCL laxity	Coronoid fracture malunion subluxation	MCl + LCL reconstruction ant capsular repair
P11	Terrible Triad	Severe global fibrosis, tissue retraction	Coronoid defect, subluxation	MCL + LCL reconstruction ant capsular repair, UN transposition
P12	Terrible Triad	Severe ant contracture ligament tear	Coronoid displacement, HO Class I	MCL + LCL reconstruction, ant capsular repair
P13	Terrible Triad	Severe global contracture, tissue scarring	Humero-ulnar tissue subluxation, HP Class II	MCL + LCL reconstruction, ant capsular repair UN transposition
P14	Elbow Dislocation	Severe ant capsular contracture	Minor periarticular calcifications	UN transposition
P15	Elbow Dislocation	Moderate global periarticular fibrosis	Concentric joint no, HO	None (core procedure only)
P16	Elbow Dislocation	Severe post capsular contracture	Minor olecranon osteophytes	UN transposition
P17	Olecranon Fr	Moderate post-capsular contracture	Retained hardware Post impingement	V-tricepsplasty HW removal
P18	Olecranon Fr	Moderate global Periarticular fibrosis	Retaining tension band wiring	HW removal
P19	Neglected RH Fr	Severe lateral contracture, LCL laxity	Severe radiocapitellar arthritis	RHA, LCL reconstruction V-tricepsplasty, UN transposition
P20	Neglected RH Fr	Severe lateral contracture LCL tear	Radiocapitellar arthritis, loose bodies	RHA, LCL reconstruction V-tricepsplasty

* Note: All 20 patients underwent the standardized core procedure (Anterior and Posterior Arthrolysis + Triceps Tenolysis). Abbreviations: Ant, anterior; Post, posterior; Fr, fracture; HO, heterotopic ossification; HW, hardware; UN, ulnar nerve; MCL, medial collateral ligament; LCL, lateral collateral ligament; RHA, radial head arthroplasty; RH, radial head.

## Data Availability

The data presented in this study are available in references [1,2,3,4,5,6,7,8,9,10,11,12,13,14,15,16,17,18,19,20,21,22,23,24,25,26,27,28,29,31].

## Abbreviations

The following abbreviations are used in this manuscript:

MEPS	Mayo Elbow Performance Score
DASH	Disabilities of the Arm, Shoulder and Hand
VAS	Visual analog scale
ROM	Range of motion
LCL	Lateral collateral ligament
MCL	Medial collateral ligament
